# Developing a theoretical evolutionary framework to solve the mystery of parturition initiation

**DOI:** 10.7554/eLife.58343

**Published:** 2020-12-31

**Authors:** Antonis Rokas, Sam Mesiano, Ortal Tamam, Abigail LaBella, Ge Zhang, Louis Muglia

**Affiliations:** 1 Department of Biological Sciences, Vanderbilt University Nashville United States; 2 Department of Reproductive Biology, Case Western Reserve University and Department of Obstetrics and Gynecology, University Hospitals of Cleveland Cleveland United States; 3 The Shraga Segal Department of Microbiology, Immunology and Genetics, Faculty of Health Sciences, Ben Gurion University Beer Sheva Israel; 4 Division of Human Genetics, Cincinnati Children’s Hospital Medical Center and Department of Pediatrics. University of Cincinnati College of Medicine Cincinnati United States; 5 Burroughs Wellcome Fund, Research Triangle Park Durham United States; Max Planck Institute for Developmental Biology Germany; Max Planck Institute for Developmental Biology Germany

**Keywords:** pregnancy, prematurity, parturition, preterm birth, evolution, eutherian mammals

## Abstract

Eutherian mammals have characteristic lengths of gestation that are key for reproductive success, but relatively little is known about the processes that determine the timing of parturition, the process of birth, and how they are coordinated with fetal developmental programs. This issue remains one of biology's great unsolved mysteries and has significant clinical relevance because preterm birth is the leading cause of infant and under 5 year old child mortality worldwide. Here, we consider the evolutionary influences and potential signaling mechanisms that maintain or end pregnancy in eutherian mammals and use this knowledge to formulate general theoretical evolutionary models. These models can be tested through evolutionary species comparisons, studies of experimental manipulation of gestation period and birth timing, and human clinical studies. Understanding how gestation time and parturition are determined will shed light on this fundamental biological process and improve human health through the development of therapies to prevent preterm birth.

## The challenge: Optimizing parturition

**Figure 1.**
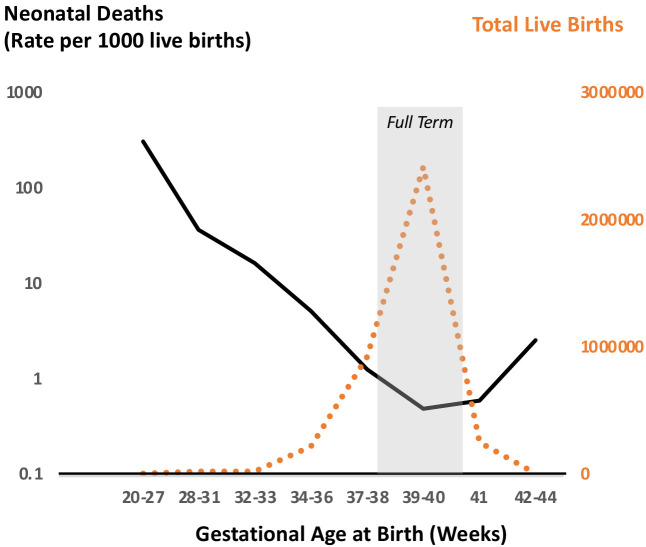
Neonatal deaths as a function of gestational age at birth for singleton pregnancies in the US in 2015. Data reflect outcomes of 3,849,557 pregnancies demonstrating optimal survival between 38 and 40 weeks of completed gestation. This optimal survival time coincides with the peak of birth timing during pregnancy as shown by number of total live births. Drawn from data in JAMA Pediatr. 2018 Jul; 172(7): 627–634 (Ananth et al., 2018).

The timing of parturition (the process of birth) is critical for survival of the neonate and the infant’s future health trajectory (Goldenberg et al., 2008; Howson et al., 2009; Liu et al., 2015; Muglia and Katz, 2010). This time in normal healthy pregnancy is referred to as term and in humans occurs at 37–42 completed weeks of gestation; the majority of spontaneous births in singleton human pregnancies take place during this window. Furthermore, neonatal death (death in the first 28 days of life) is lowest, and thus survival and fitness are highest, for neonates born after 37 and before 42 completed weeks of gestation (Figure 1). Reflecting these survival relationships, the current definition of a ‘full term’ pregnancy is now 39–40 weeks of completed gestation (https://www.acog.org/clinical/clinical-guidance/committee-opinion/articles/2013/11/definition-of-term-pregnancy).

The observation that the peak of birth timing in women coincides with the trough of neonatal mortality suggests that developmental and physiological mechanisms that optimize the timing of parturition have likely been under strong selection through evolution. Similarly, genetic polymorphisms in mothers and newborns that contribute to optimization of parturition timing may too be under the influence of selection. Of course, the optimal time for birth is also influenced by an organism’s life history and ecology, such as litter size, maternal habitus, circadian and seasonal activity patterns, and social and environmental exposures, all of which vary widely across mammals. Therefore, it is not surprising that much diversity exists in the physiology of pregnancy and birth timing among mammals. This is evidenced by the observation that genes associated with reproduction exhibit high levels of sequence divergence among eutherian mammals, similar to those associated with adaptive immune system development and function (Chimpanzee Sequencing and Analysis Consortium, 2005; Waterston et al., 2002).

It is plausible, therefore, that the biological processes that determine the timing of human parturition are specific for the *Homo* lineage. This lineage specificity would explain why cross-species observations, although revealing some principles of birth timing, have not significantly advanced understanding of human birth timing. One reason for this evolutionary divergence in strategies between humans and other species may be the unique increase in brain size in humans, even in comparison to other primates, over the last few million years and the physical or metabolic constraints encountered because of it (Pavličev et al., 2020). This dearth of knowledge has clinical and sociological implications because it has slowed advances in development of therapies to prevent preterm birth (i.e. birth before the 37th week of gestation), which is the leading cause of death for newborns (Howson et al., 2012) and children under 5 years of age (Liu et al., 2015). For infants that survive past 5 years, preterm birth leads to increased incidence of lifelong health impairments, such as growth, neurological, and sensory deficiencies (Committee on Understanding Premature Birth and Assuring Healthy Outcomes Board on Health Sciences Policy, 2007). Despite the recognized importance of term pregnancy for optimizing reproductive fitness and lifelong health, and the enormous cost of preterm birth, understanding of the molecular signals that control human birth timing remains scant (Committee on Understanding Premature Birth and Assuring Healthy Outcomes Board on Health Sciences Policy, 2007). Importantly, not all populations are equally impacted by preterm birth, with African American women experiencing an especially increased burden (Collins and David, 2009; Kistka et al., 2007a). Social determinants of health disparities, and the consequences of racism, (Slaughter-Acey et al., 2016; Murrell, 1996) must be incorporated into efforts to improve outcomes for all populations. In this review, we focus on the biology of birth timing determination, recognizing that this biology is strongly influenced by social and environmental parameters.

## Six core principles governing the evolution of parturition

**Figure 2.**
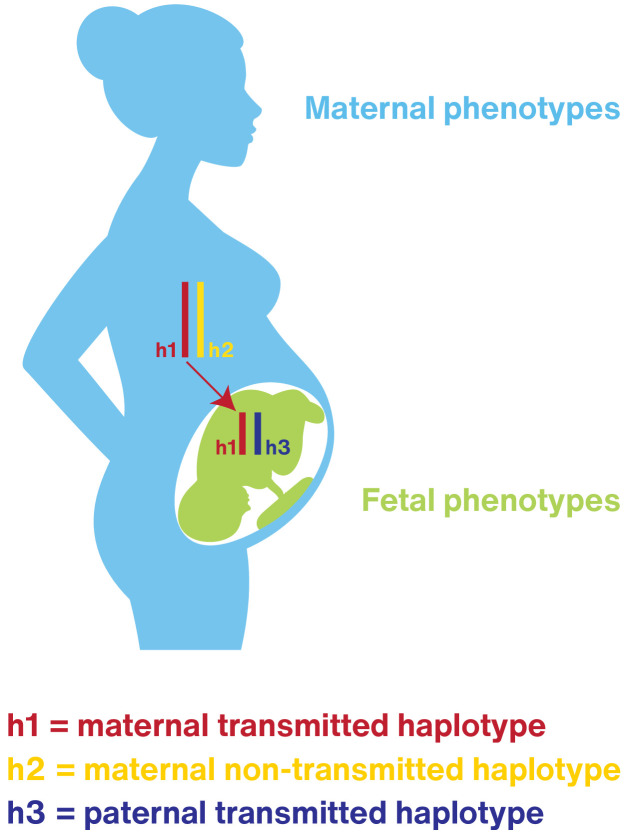
Pregnancy-associated traits are influenced by both maternal and fetal genetics. One framework for studying the maternal and fetal genetic effects in pregnancy is to consider the mother/fetus pair as a unit with three haplotypes, h1, h2, and h3. The maternal-fetal compound genome of pregnancy genetically contributes to maternal phenotypes (blue) through the actions of the maternal transmitted (h1, red) and non-transmitted haplotypes (h2, yellow). Fetal phenotypes are genetically determined by the maternal transmitted (**h1**) and the paternal transmitted (**h3**) haplotypes which may display parent of origin effects. The maternal environment, shaped by h1 and uniquely by h2, also influences pregnancy outcomes including pregnancy duration through maternal effects.

One approach to addressing our knowledge gap on mammalian parturition is to develop a conceptual evolutionary framework. Birth timing is a key event in the reproductive cycle of eutherian mammals and as such is likely subject to past and ongoing selective pressure to optimize reproductive efficiency with respect to organisms’ life histories, ecologies, and social and environmental exposures. Although these selective pressures have given rise to the diversity in the physiology of pregnancy and birth timing that is observed among mammals, a conceptual framework to describe the processes common to all eutherian mammals can be developed based on six core principles:

1. pregnancy is a temporary state that imparts survival risk for the female and her fetus/neonate and as such must eventually end through expulsion or resorption of the conceptus;
2. retention of the conceptus requires that the parturition process is dormant or actively blocked for a specific amount of gestation time. The steroid hormone progesterone is a conserved endocrine signal across viviparous species that serves this purpose (Rothchild, 2003; Young et al., 2010);
3. parturition may be initiated by multiple pathways, some of which are part of the normal timing process and provide complementary, partially overlapping mechanisms, while others (e.g. pathways associated with response to infection) may override pregnancy maintenance signals to provide fail-safe mechanisms that serve the survival interests of the mother (and her future reproductive potential) and the fetus/neonate (Rothchild, 2003; Young et al., 2010; Challis, 1980; Challis and Mitchell, 1981; Mitchell and Taggart, 2009);
4. each mammalian species has evolved its own strategy for parturition timing, which may or may not be conserved in other species. Compared to other organs or systems, reproductive organs (e.g. uterus and placenta) are more recently evolved; new functions or novel cell types in these organs are thought to have evolved from existing tissues by rewiring existing genetic regulatory networks; (Kin et al., 2015; Lynch et al., 2011; Lynch et al., 2004; Wagner and Lynch, 2005; Wagner and Lynch, 2010; Griffith and Wagner, 2017; Plunkett et al., 2011; Wildman et al., 2006; Zhang et al., 2020)
5. pregnancy-associated traits, including parturition, evolved ‘on’ pre-existing cardiovascular, metabolic, and immune systems, and the reproductive strategy coordinates with these systems (Bainbridge, 2014). At the genetic level, these traits are influenced by many genetic variants with interactions between genes. The polygenic control of pregnancy-associated traits may facilitate adaptation and evolutionary robustness (Gibson and Lacek, 2020). The evolution of pregnancy-associated traits may have diverse effects on other systems and conversely, should be constrained by genetic correlations with other traits; and
6. pregnancy-associated traits, including parturition, are influenced by both maternal and fetal genomes and parental environmental effects (Figure 2). The involvement of the maternal and fetal genomes and the parental environmental and epigenetic cross-generational effects have profound evolutionary implications and have likely experienced both conflict and coadaptation (Miska and Ferguson-Smith, 2016; Badyaev and Uller, 2009). For example, as evidenced by the consequences of the Dutch famine, subsequent generations’ pregnancy outcomes were adversely affected (Roseboom et al., 2011).

## Viviparity

The core principles listed above are shared across eutherian mammals due, in part, to the ancestral evolution of viviparity—to understand the evolution and diversity of parturition we must uncover the evolutionary substrate upon which it was formed. The hallmark traits of eutherian viviparity are: (1) retention and gestation of the fertilized egg(s) in the female reproductive tract (uterus); (2) development of a placenta that facilitates the transfer of oxygen and nutrients from the maternal compartment to the conceptus, and (3) delivery of the fetus and its placenta via the process of parturition at a specific stage of gestation. Each trait is critical for the success of viviparity, and therefore has likely been under strong selective pressure to optimize reproductive fitness (Mitteroecker et al., 2017; Mitteroecker et al., 2016). The course of evolution among mammalian viviparous species, which has been influenced by both selective and neutral processes, has produced considerable variation in diverse reproductive traits, such as in the number of ovulated and retained eggs, the structure of the uterus, the anatomy of the maternal-placental interface, the structure and function of the placenta and its effect on maternal physiology, the rate of embryo/fetal development and functional maturity of the neonate, and the signals that lead to parturition (Rothchild, 2003; Swaggart et al., 2015; Plant and Zeleznk, 2015).

Phylogenetic analyses suggest that viviparity originated from oviparity (expulsion of the egg soon after fertilization and its independent development in the external environment) in at least 150 lineages, including in mammals, fish, and reptiles (Blackburn, 2015). The evolution of viviparity with the establishment and maintenance of prolonged gestation characteristic of eutherians may inform potential models of parturition initiation. The evolution of invasive placentation in eutherians that allowed for prolonged intrauterine gestation differentiates them from marsupials, which develop very transient placentas, and monotremes, which are oviparous (Wildman et al., 2006; Chavan et al., 2016). Elegant studies by Wagner and colleagues have shown that eutherian implantation evolved from the marsupial attachment reaction, with similar proinflammatory gene expression signatures (Griffith et al., 2017). An innovation in eutherians was the down-regulation of inflammation after embryo attachment to the maternal endometrium reflected in their endometrial stromal fibroblast transcriptomic profiles in comparison with marsupials (Kin et al., 2016). Additional anti-inflammatory mechanisms likely evolved in the fetus or placenta as suggested by the expansion of the tripartite-motif family of genes and their ability to finely tune inflammation during placentation (Zhang et al., 2020). Considering these evolutionary innovations that allowed for the establishment of eutherian gestational prolongation suggests two potential different strategies that natural selection could influence to determine birth timing. One strategy would be to suppress the specific anti-inflammatory signals that allowed sustained placentation to be established. The other strategy would be for selection to act on other molecular targets that would overcome the anti-inflammatory signals that down-regulated the proinflammatory signals requisite for the attachment reaction. The genes mediating these effects across species would themselves be subject to the diverse evolutionary pressures as suggested by recent genome wide association studies for human gestational duration (LaBella et al., 2020).

## Birth timing

Parturition, and especially its timing, is a mission-critical event for the success of mammalian viviparity. Normal parturition occurs at a specific time referred to as term, when the fetus is sufficiently mature to survive as a neonate, and the mother is able to provide care for the neonate’s nutrition, protection and physiologic stability, while preserving her own fitness. Timing mechanisms for parturition have likely been selected to optimize reproductive fitness based on benefits to the mother and fetus for the current pregnancy, and benefits to the mother’s survival and future reproductive potential (note that these two are not always aligned). One important general association with birth timing is its correlation with body size at birth (Phillips et al., 2015). This association may reflect energy utilization particularly due to the developing fetal brain (Dunsworth et al., 2012) or physical constraints such as infant size related to the size of the birth canal (Rosenberg and Trevathan, 2002; Rosenberg and Trevathan, 2001).

Studies utilizing concordance in birth timing in offspring of twins, or family-based segregation or epidemiology, suggest that 30–40% of the variation in human birth timing is due to genetic factors, and these reside largely in the maternal genome (Kistka et al., 2008; Plunkett et al., 2009). The ‘compound genome’ of pregnancy is unique when considering the maternal-fetal unit during gestation, consisting of DNA composition of 3 distinct haplotypes. How these unique or shared haplotypes interact to produce the 30–40% variation in human gestation length is only beginning to be explored (Zhang et al., 2015; Zhang et al., 2018; Figure 2). The remaining 60–70% of the variation is thought to arise from environmental influences of uncertain origin. These may include nutrition, infectious disease, health behavior, and social circumstances/stress. Furthermore, recent genomic investigations have demonstrated a causal relationship between single nucleotide polymorphisms (SNPs) that associated with adult height and gestation length in humans such that taller maternal height and its genetic determinants leads to longer gestation (Zhang et al., 2015). Other maternal traits, such as fasting blood glucose and blood pressure, have also been shown to causally determine length of gestation or fetal size at birth in humans (Chen et al., 2020). Recent ability for GWAS comparison of those loci that uniquely determine gestational duration with those that have been associated with birth weight provide an interesting avenue moving forward to disentangle the underlying genetics responsible for the relationship of gestational duration and birth weight (Early Growth Genetics (EGG) Consortium et al., 2018; Zhang et al., 2017).

Do species other than humans exhibit a significant frequency of preterm birth? This question is difficult to answer since preterm birth in humans is an arbitrary definition. If we simply scale the assignment of preterm birth based upon duration of term gestation (37 out of 40 weeks, or 92.5% of gestation) then other eutherian mammals also experience preterm birth (Phillips et al., 2015). Frustratingly, the most commonly studied, genetically tractable animal model, the mouse, does not appear to exhibit spontaneous preterm birth (Phillips et al., 2015). Does this reflect laboratory selection in strains currently utilized or a fundamental trait of rodents? This question merits investigation. Other non-traditional animal models that demonstrate spontaneous preterm birth, such as mammals used in livestock (Nielsen et al., 2016; Fthenakis et al., 2012; Scott et al., 2008; Asher, 2007), could be utilized to investigate preterm birth and low birth weight pregnancies in the future.

## Maintaining pregnancy

Pregnancy is the physiologic state in eutherian mammals in which a fertilized egg is retained and gestated in the cavity of the uterus. For pregnancy to be maintained, the smooth muscle of the uterine wall (the myometrium) remains relaxed, quiescent, and grows and distends to accommodate growing conceptus, whereas the uterine cervix remains rigid and closed. In addition, maternal physiology adapts to provide nutrients and oxygen to the developing fetus. Remarkably, a conserved trait in all viviparous mammals examined so far is that the establishment and maintenance of pregnancy are dependent on the steroid hormone progesterone (P4) (Corner and Allen, 1929; Csapo, 1956). As its name implies, P4 is a pro-gestation hormone. It promotes myometrial quiescence, cervical closure, immunologic tolerance at the maternal-fetal interface and, importantly, blocks parturition. Remarkably, the embryo-retaining actions of P4 have been conserved through multiple viviparous lineages, including lizards (Jones, 2017).

P4 is synthesized by enzymatic modification of cholesterol. At the time pregnancy is established, P4 arises from the corpus luteum (CL) of the ovary. Some species, such as rodents, maintain ovarian P4 production as the sole source of P4 to maintain pregnancy, and parturition is initiated by loss of P4 due to CL regression (Ratajczak et al., 2010). In rodents, regression of the maternal CL is induced by prostaglandin F2α (PGF_2α_) produced by the uterine endometrium acting on its receptors on cells in the CL. Thus, term parturition in rodents is induced by PGF_2α_ production in the endometrium. In other species, such as sheep and primates, the source of P4 for pregnancy maintenance shifts from the CL to the placenta (Mesiano et al., 2014; Tuckey, 2005). In humans, this occurs at approximately the 10th week of gestation. In pregnancies associated with fetal deficits, and inadequate function of fetal-placental-maternal signaling, miscarriage commonly occurs at this ovarian-placental progesterone transition point (Baird, 2009). In sheep, as in rodents, maternal circulating P4 declines acutely (due to decreased P4 production by the placenta), prepartum to promote parturition (Liggins et al., 1977). This, however, does not occur in primates. Although maternal blood P4 levels vary widely among anthropoid primates (New World monkeys, Old World monkeys, and apes), a conserved trait in this group is that parturition occurs without systemic P4 withdrawal (Ratajczak et al., 2010). Nonetheless, disruption of P4 signaling by treatment with the P4 receptor (PR) antagonist, RU486, induces parturition in all species examined so far, including humans (Garfield and Baulieu, 1987). One explanation for this inconsistency is that primate parturition involves a functional P4 withdrawal whereby target cells in the uterus become refractory to the P4 block to labor. Studies suggest that this occurs by multiple mechanisms including local metabolism of P4 to an inactive form (i.e. tissue or target cells P4 withdrawal) (Nadeem et al., 2016) and alteration in PR transcriptional activity in uterine target cells (Mesiano et al., 2014; Wu and DeMayo, 2017).

## Initiating parturition and ending pregnancy

**Figure 3.**
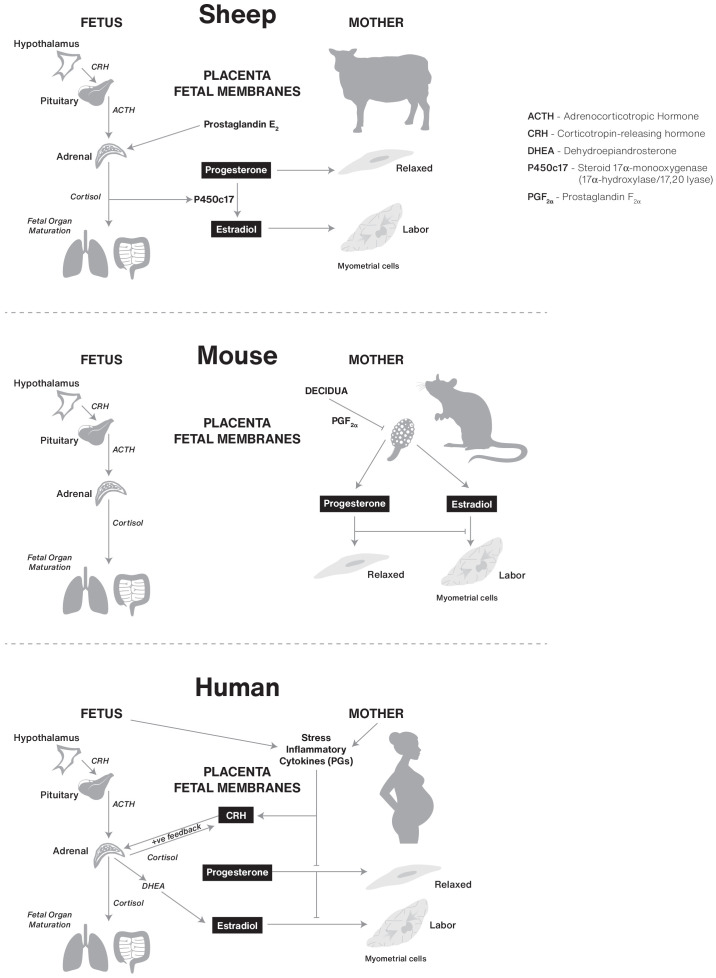
Comparative biochemical strategies for initiating parturition in sheep, mouse, and human species. The steroids estrogen and progesterone are conserved components, but the tissues involved and superimposed innovations, such as CRH in humans, likely result in divergence of final pathways for birth.

The control of human birth timing remains enigmatic, largely because of our limited ability to precisely predict its occurrence based upon absolute days of gestation. In contrast, the timing of birth in other species, exemplified by sheep and mice, is predictable and the underlying regulatory signals have been defined. For example, pioneering studies by Liggins, 1974 demonstrated that parturition in sheep is initiated by increased activity of the fetal hypothalamic-pituitary-adrenal axis to produce a surge of cortisol that decreases P4 production by the placenta leading to systemic P4 withdrawal that initiates parturition (Figure 3 and Table 1). Importantly, the cortisol surge also induces functional maturation of fetal organ systems, and especially surfactant production by the lungs, in preparation for life outside the uterus. Disruption of the fetal HPA inhibits ovine parturition and treatment with cortisol into the fetus initiates parturition. Thus, the ovine mechanism for birth timing is solely controlled by cortisol produced by the fetal HPA axis that coordinates the timing of birth with fetal maturation and readiness to exist as a neonate.

**Table 1.** Comparative analysis of P4 source, maintenance, and role in parturition initiation for rodent, sheep, and human pregnancy.

	P4 Source	Pregnancy Maintenance	Parturition Initiation	Mechanism of Initiation
Rodent	CL	P4	Systemic P4 withdrawal	Endometrium production of PGF2α. CL regression.
Sheep	CL transition to Placenta	P4	Systemic P4 withdrawal	Increased activity of the fetal HPA axis. Increase of cortisol. Decrease P4 production.
Human	CL transition to Placenta	P4	No systemic P4 withdrawal (tissue or target cell resistance)	Potential contributors: Change in progesterone receptor isoforms. Change in PR cofactors. PR post translational modifications. Local metabolism of P4.

In contrast, glucocorticoids do not induce labor in mice or humans. In mice, parturition is initiated after upregulation of endometrial cyclooxygenase (COX)–one encoded by *PTGS1*, which subsequently generates prostaglandin F2α (Gross et al., 2000; Gross et al., 1998; Sugimoto et al., 1997). PGF2α from the endometrium then acts on the corpus luteum of the murine ovary to induce luteolysis, a fall in serum progesterone and the induction of contractile proteins in the uterine myometrium (Figure 3 and Table 1).

Cortisol plays a key role in promoting fetal organ maturation in humans, however, parturition in women is not initiated by fetal HPA activity. Although the work of Liggins and colleagues in sheep was groundbreaking for understanding hormonal pathways that may promote parturition, the specific pathways operating in the sheep do not appear to operate in the same way in women (Liggins et al., 1977). The ancestral initiating signal for determining the time for mammalian birth remains elusive and as a consequence of this knowledge gap, rational effective interventions to prevent preterm labor and preterm birth in humans have been limited. Nonetheless, the work of Liggins and colleagues in sheep led to the widespread clinical use of synthetic glucocorticoid therapy to promote fetal lung maturation in women that are either experiencing preterm labor and that are at risk for preterm birth, which significantly improved survival of preterm neonates.

Beyond the glucocorticoid signal, several labor-permissive or promoting signals have been identified across mammals. Foundational among these are estrogens (mainly estradiol) that are also steroid hormones derived from cholesterol, whose abundance increases at the end of gestation in most species and act to augment uterine contraction by inducing the expression of genes in uterine smooth muscle cells encoding factors that increase contractility and excitability. For example, in the rat and mouse estradiol induces the expression in myometrial cells of *GJA1* that encodes connexin-43 a major subunit of intracellular tight gap junctions that facilitate contractile synchrony between myometrial cells, and *OXTR* that encodes the oxytocin receptor and increases myometrial sensitivity of the uterotonic actions of oxytocin., *PTGFR* encodes the prostaglandin FP receptor and *PTGS2* that encodes cyclooxygenase-2 (COX-2) and generates critical parturition proinflammatory prostanoids, such as PGE2 and PGF2alpha, that induce cytokines such as interleukin-1 that are also pro-labor (Keelan et al., 2003; Ratajczak and Muglia, 2008; Smith, 2007).

Uterine tissue-level inflammation is associated with normal term parturition across mammals. However, less clear is whether proinflammatory signals are involved in the parturition initiation process from those species that do not undergo luteolysis and systemic progesterone withdrawal or act to facilitate or accelerate the process once initiated. In primates, the inflammatory cascade is apparent but may come after the proximal initiator of parturition (Liu et al., 2019). For example, recent genome-wide association findings in humans identify the fetal pro-inflammatory locus encompassing the IL-1 family on chromosome two as significantly associated with gestational duration. Interestingly, the association with prolonged gestation, not preterm birth, suggests that the locus contributes to promoting the birth process downstream of the initiating signals; thus, initiating and facilitating signals may be distinct (Liu et al., 2019).

## Post-term birth

While the leading cause of perinatal death is preterm birth, postterm birth (delivery at gestational age more than 294 days or 42 full weeks of completed gestation) also places the newborn at higher risk for perinatal mortality and neonatal acute and long-term adverse health effects (Kortekaas et al., 2015; Seikku et al., 2016; Linder et al., 2017; Figure 1). Viewing preterm and post-term birth as the two tails of the gestational age distribution, it is reasonable to hypothesize that they may reflect opposite regulatory directions of action of the same pathophysiological pathways in at least some circumstances. Therefore, understanding the mechanisms for postterm birth might potentially yield insights into underlying mechanisms of normal and preterm parturition as well.

As in preterm birth, recent studies have shown potential genetic contributions to postterm birth, such as maternal recurrence and familial risk, ethnicity and common variant associations (Morken et al., 2011; Kistka et al., 2007b; Schierding et al., 2018; Oliver et al., 2018) For example, a mother born postterm has a 49% higher relative risk to have a postterm pregnancy, whereas a father delivered postterm contributes 23% increased risk. The risk is even higher when both the mother and father were born postterm (Morken et al., 2011). In addition, women with postterm delivery in their first pregnancy have significantly higher risk of recurrence in subsequent pregnancies (Morken et al., 2011). A recent genome-wide association study of prolonged gestation in the Northern Finland found a significant association with postterm birth in an intronic region within the *ADAMTS13* gene, also known as von Willebrand factor-cleaving protease, that is involved in blood clotting (Schierding et al., 2018). As in preterm birth, there is also evidence for prolonged gestation duration in humans associated with geographic/genetic ancestry. For example, women of Somali ancestry have a significantly increased risk for prolonged gestation even after adjustment for potential confounding factors (Oliver et al., 2018).

## Developing a theoretical evolutionary framework to gain insights into the biology of parturition

**Figure 4.**
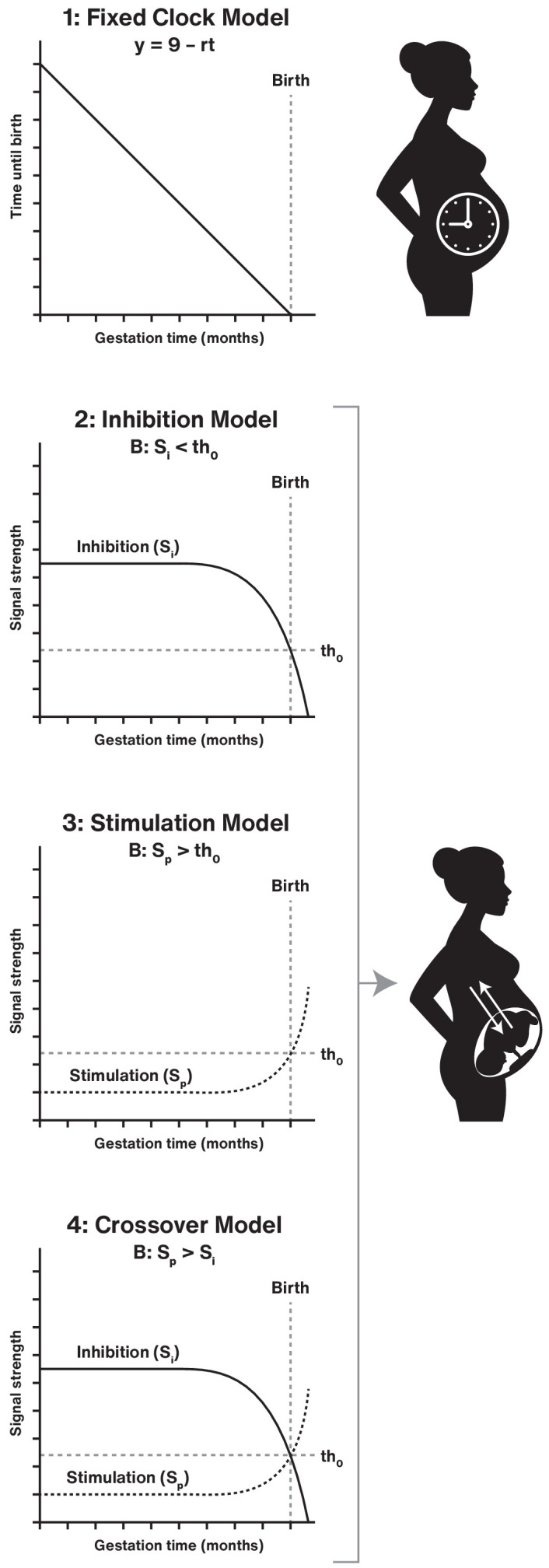
Testable theoretical models for determining the onset of parturition in eutherian mammals. Model one represents a **fixed clock** in the mother or fetus to determine when birth happens (y = 0). The precise day is determined by a physiological rate constant (**r**) that determines when, for a given woman, birth would occur. The time (**t**) is measured in months in this representation. Model two reflects a parturition **inhibition** signal (S_i_) that must go below a threshold (th_0_) for birth (**B**) to occur. Model three alternatively proposes a parturition **stimulation** signal (S_p_) that must exceed a threshold for birth to occur. Model four displays that both S_i_ and S_p_ are involved in determining the timing for birth, and parturition occurs as a **crossover** when S_p_ >S_i_.

The insights derived from recent evolutionary genomic analyses of mammalian pregnancy and genome-wide analyses of human populations and the identification of genes involved in gestation length described above showcase both the depth of knowledge on mammalian parturition as well as why its mechanisms largely remain mysterious. In particular, our knowledge of parturition mechanisms in humans is complicated by both the likelihood of lineage-specific mechanisms and our limited ability to experimentally interrogate human pregnancy due to ethical and technological considerations. While currently available data do not reveal a complete picture of the initiation of term and preterm parturition, they do allow us to develop a conceptual evolutionary framework upon which to test future hypotheses. As a first step toward establishing an evolutionary framework for the timing of mammalian parturition, we outline four theoretical models for how the onset of parturition is determined in a general way around core principles (Figure 4). These four models provide examples of testable hypotheses.

It is important to note that the four models aim to explain the maintenance of pregnancy and initiation of parturition, which should be distinguished from the contractile mechanism to expel the fetus. The contractile mechanism is downstream of the initiation signal, is likely to involve redundant uterotonic pathways, and is probably going to be much more challenging to target effectively for preterm birth prevention compared to initiator pathways (Goldenberg et al., 2008). We encourage researchers to test these models or other models with empirical data to determine detailed mechanisms, confirming, refining or refuting their predictions, including how social stress, racism, and health behaviors affect risk for preterm birth.

### Fixed clock model

The onset of parturition is determined by the mother or the fetus based upon a hard-wired clock. The fixed gestation clock hypothesis posits a time-measuring or counting process that accrues from the point of fertilization. In this model, parturition begins when a certain time or count is achieved (Menon et al., 2016). The timing mechanism may reside in the conceptus (e.g. the number of cell divisions after syngamy) and/or involve an isochronous measure linked to circadian signals or photoperiod. Genetic and epigenetic polymorphisms between individuals or different environmental exposures may modify the clock, leading to variation in gestation length and timing of parturition.

The fixed clock model would be selectively advantageous if birth timing is linked to the fetal developmental program. To survive as a neonate the fetus must have the capacity to establish and maintain homeostasis immediately after delivery. The principal cause of neonatal mortality and morbidity in preterm infants is organs system immaturity. The reproductive cost of neonatal immaturity would have exerted strong selective pressure to favor traits the ensure that the fetus is not born before it is ready for life outside the uterus.

Is there evidence suggesting that human birth timing is linked to the fetal development program? Support for linkage of the two processes comes from sheep, where parturition is trigged exclusively by a surge of cortisol produced by the fetal hypothalamic-pituitary-adrenal (HPA) axis late in pregnancy. Importantly, the cortisol also stimulates functional maturation of fetal organ systems, especially the lungs, gastrointestinal tract and liver, thus ensuring the newborn lamb is equipped for life outside the uterus. However, studies in multiple species show that although the effect of fetal cortisol to promote organ system maturation appears to be conserved across species (e.g. prepartum glucocorticoid therapy to accelerate fetal lung development in cases of threatened preterm birth has significantly improved outcomes for human preterm infants), its role as a driver of parturition is not conserved (Figure 3 and Table 1).

### Parturition block model

Retention of the conceptus in the uterus is a hallmark trait of eutherian mammals and requires that the uterine tissues accept and accommodate the developing conceptus for the gestation time needed to complete the fetal development program. The parturition block model posits that a blocking agent or system dominates for most of pregnancy to actively prevent parturition, and that labor and delivery ensue when the block is removed. This implies that if not for the block, the uterus would expel its contents. Interestingly, uterine emptying is not limited to parturition. Large uterine fibroids can be spontaneously expelled through a process that includes myometrial contraction and dilation of the cervix (De Cure et al., 2013; Sagoo et al., 2015). In cases of uterine fibroid expulsion, the fibroid mass is necrotic and patients exhibit signs of intrauterine infection and inflammation. It is plausible that the same inflammation-associated mechanism could operate at parturition to overcome the block to labor.

Existence of a parturition block was first recognized in the late 1800s from observation that pregnancy in rabbits is dependent on a factor secreted from the maternal corpus luteum and that removal of the CL initiated parturition at all stages of pregnancy. The pro-gestation parturition blocking factor was eventually identified to be Δ5-keto-steroid and named progesterone (P4) (Allen, 1930; Allen et al., 1935; Allen and Reynolds, 1935; Allen and Wintersteiner, 1934). Studies in animal models subsequently showed that P4 prevents ovariectomy-induced parturition, and that withdrawal of P4 alone induces parturition. It is now clear that P4 is essential for the establishment and maintenance of pregnancy and that removal of its block to parturition is a conserved event in the physiology of mammalian birth timing [for review see Csapo, 1956; Corner, 1942]. Diversity exists in both the source of P4—maternal CL or placenta—to maintain pregnancy, and the mechanism for its withdrawal—systemic withdrawal or functional desensitization of uterine target cells—to promote parturition (Ratajczak et al., 2010; Young et al., 2010).

The P4 block model posits that birth timing is determined by factors that induce actual or functional P4 withdrawal. P4 is thought to maintain pregnancy by exerting anti-inflammatory effects on the uterine tissues, thus preventing inflammation-associated uterine emptying. In this scenario, the gravid uterus is poised and prepared for parturition throughout pregnancy but is restrained by an anti-inflammatory P4 block. Diversity exists in upstream signals to induce withdrawal of the P4 block. The P4 block to labor may diminish with advancing gestation under the control of a clock mechanism until a threshold is reached below which the parturition cascade is initiated. The system may function in a stochastic manner – the probability of parturition increases with gradual decline of the P4 block. It is also possible that multiple up-stream signals for parturition converge to induce P4 withdrawal.

In most eutherian mammals, parturition is associated with a systemic decrease in maternal P4 levels due to regression of the CL or the induction of enzymatic activity in placental cells that mitigates P4 secretion. This, however, does not occur in humans and other anthropoid primates; instead, parturition occurs with the uterine tissues exposed to high levels of P4 (Ratajczak et al., 2010). The suggested original ancestral state for modulation of circulating maternal P4 signaling leading to parturition is one of systemic circulating P4 decrease (withdrawal) as revealed in phylogenetic analyses based upon outgroup comparison with hoofed animals and carnivores (Nnamani et al., 2013). This suggests that human and primate parturition involves some form of P4-block override. Indeed, disruption of P4 signaling via the nuclear P4 receptors (PRs), increases uterine contractility, promotes cervical softening and, in most cases, induces the full parturition cascade. This argues that the P4 block to labor is mediated by the nuclear P4 receptors (PRs) and that modulation of PR signaling may be an alternative mechanism for P4 withdrawal when circulating P4 levels are not reduced.

The parturition block model reflects the balance between maternal and fetal/paternal genetic interests in the pregnancy condition. Natural selection would favor a conceptus that can maintain pregnancy and extract resources form the mother for the longest amount of time. Studies of imprinted genes suggest that this trait is in part conferred by paternally imprinted genes. In contrast, the interests of the mother include surviving the current pregnancy and maintaining her reproductive competence for future pregnancies and caring for current dependent offspring. In this context, it would be logical that a parturition block signal would be coming from the fetus. However, the block cannot be absolute and would need to be counterbalanced by maternal signal(s) to stimulate parturition when maternal interests and/or fetal viability are compromised. Thus, a fetal-based parturition block would be expected to eventually diminish and/or be overcome by a maternal-based parturition stimulator signal. Indeed, a conserved trait in eutherian mammalian species is that parturition is promoted by withdrawal of the P4-block to labor, suggesting that the stimulatory signal(s) for parturition converge on nullifying the P4 block.

### Parturition stimulation model

This model posits that the labor state is actively induced by acute or gradually accumulating signals. The signals may be linked to the size of the conceptus and the amount of uterine wall and cervical distention (Rosenberg and Trevathan, 2002), stressors of fetal or maternal origin when fetal growth rate exceeds energy availability (Dunsworth et al., 2012), signals derived from the maturation of fetal organ systems (Condon et al., 2004; Mendelson, 2009), or signals derived from the maternal stress load. Parturition is associated with uterine tissue-level inflammation especially at the chorion-decidua interface. Clinical studies show that human parturition can be induced by inflammation/infection and that ~ 50% of preterm births are associated with intrauterine inflammation or infection (Goldenberg et al., 2008; Goldenberg et al., 2000; Romero et al., 2006). Further, animal studies show that pro-labor inflammatory cytokines and bacterial infection induce parturition (Hirsch and Muhle, 2002; Elovitz and Mrinalini, 2004). It is likely, therefore, that one element of the parturition stimulation model is maternal/fetal inflammatory signaling. In this context it is notable that P4/PR action is thought to block parturition by exerting anti-inflammatory effect on the uterine tissues. Recent studies suggest that inflammatory stimuli modulate PR signaling in human myometrial cells to induce functional P4/PR withdrawal (Amini et al., 2016). This suggests that the P4/PR anti-inflammatory activity can be overcome by inflammatory stimuli. Although counterintuitive, such a mechanism could operate if an inflammatory stress threshold exists, above which the P4/PR block is removed. A threshold-limited mechanism that balances pro-gestational actions of the P4/PR block against the magnitude of stress-related parturition signals would provide room for the adaptation of multiple maternal/fetal stress-related pathways to induce parturition.

### Crossover combined model

This model posits that a combination of restraining and promoting signals leads to parturition when a crossover threshold is reached for dominant labor-promoting actions, as suggested in a more specific manner for the relationship of P4 and inflammation signals (Brubaker et al., 2016). This model also suggests that the gestation clock, parturition block and parturition stimulatory models co-exist as part of a complementary, overlapping, and fail-safe system to control birth timing.

The importance of birth timing on species-level reproductive efficiency would have favored multiple signals for parturition depending on lineage-specific selective pressure. In this scenario natural selection would favor mechanisms promoting robustness and phenotypic stability that would include buffering by genetic networks, epigenetic canalization, and complementary, partially overlapping or redundant actions across pathways or genes as described for other developmental systems (Hallgrimsson et al., 2019). In general, the parturition mechanism attributed to a species is that which is observed to operate at term. Other mechanisms may, however, be available and operational if needed based on physiologic and environmental conditions. This concept is apparent in mice. Although term parturition in the mouse is timed by a gestation clock that causes systemic P4 withdrawal secondary to CL regression, it can also occur without systemic P4 withdrawal in response to an inflammatory challenge (Hirsch and Muhle, 2002; Hirsch et al., 1995). This suggests that multiple mechanisms for P4 withdrawal may co-exist and be invoked depending on upstream physiology. Crossover between models could be threshold-limited. Effectiveness of the P4/PR block may be gradually lost in response to a gestation clock signal or the gradual increase maternal stress load. At different gestational stages, the various mechanisms may impact the onset of partition differently. For example, the failure of the P4 block signal could cause early pregnancy loss or preterm birth, whereas a labor-promoting signal might play an important role in the fine control of the timely onset of term parturition.

### Testing the four models

Can the four proposed models be experimentally distinguished? A useful starting exercise is to determine if existing data refute one or more of the models. Significant divergence in mechanisms may exist across species, so whether one species follows a specific model cannot be completely understood by findings in another. For example, one clear piece of evidence that argues against the Fixed Maternal Clock Model comes from examining the timing of birth in cross-species hybrids. Matings between female horses and male donkeys produce mules. Under the Fixed Maternal Clock Model, the time to birth in such pregnancies would be expected to reflect the mother’s clock. However, mules are delivered on average between (11.4 months) when a horse (11.2 months) and donkey (12 months) would normally deliver (Giger et al., 1997), consistent with expectations that both maternal and fetal genomes and parental effects govern the timing of parturition. To further distinguish between maternal and fetal fixed clocks regulating birth timing, generating pregnancies where maternal and fetal gestational duration are discordant may prove revealing. One such experiment could be to produce females that are pseudopregnant for different temporal windows, and transfer fertilized embryos of different gestational ages into their uteri. This experiment would test whether the day of delivery reflects the gestational duration of the mother or the fetus. A limitation of this experiment is the potential of these pre-implantation embryos to synchronize or re-set development at the time of implantation, which could be measured by counting somite number several days into the pregnancy (Tam, 1981) or using other staging measures.

In sheep, fetal hypothalamic-pituitary-adrenal axis activation is essential for parturition and may suggest a fetal fixed clock. However, fetal adrenal activation may still be downstream of the true parturition initiating signal and represent a downstream effector pathway. Rodent pseudopregnancy, the state which arises when receptive females are mated to sterile males, lasts approximately one-half the duration of a normal, fetus-containing pregnancy (Sulila et al., 1988). This suggests that fetal signals must be contributing to pregnancy maintenance through to the normal endpoint. However, this model does not establish evidence of a fixed fetal clock to end pregnancy.

A classic physiological approach to dissecting stimulatory or inhibitory signals influencing a phenotype, such as in our Models 2–4, is to perform cross-circulation, or parabiosis, experiments. These experiments link the circulations of two animals to allow circulating factors to be shared between them. As applied to rat gestation and parturition, studies have been performed which revealed the ability of a gravid female 2 or 3 days earlier in gestation (i.e. mated later) to delay the onset of birth in the gravid female that had been impregnated earlier (Mantalenakis and Ketchel, 1966). Interestingly, gravid females 4 days earlier in gestation than the partner did not delay the onset of labor (Mantalenakis and Ketchel, 1966). This suggests an inhibitory factor from the earlier gestation animal that rises through pregnancy is capable of inhibiting parturition. These studies did not conclusively identify such a factor, but the findings are consistent with the known pattern of P4 rise during gestation and the ability of P4 supplementation to delay the onset of labor. The systemic P4 withdrawal known to cause mouse and rat parturition is thus supported by these studies (Pepe and Rothchild, 1974; Winchester et al., 2002). What this approach did not reveal was the upstream signal that initiated P4 withdrawal that arises from local prostaglandin production from the endometrium as revealed through later genetic and other physiological studies (Winchester et al., 2002). Similar parabiosis approaches have been used in guinea pigs, which do not display systemic P4 withdrawal or labor extension with P4 supplementation, which differs from rats and mice. Those studies demonstrated circulating signals that maintain uterine quiescence but did not report consequences for birth timing (Porter, 1972).

The above methods for testing the proposed models for parturition initiation, provide interesting and useful information, although each has its own limitations. We propose that applying an evolutionary, comparative framework will complement these methods, together leading to otherwise unobtainable insights. It is generally accepted that the initiator of mammalian parturition must be under strong selective pressure to optimize reproductive fitness (Rosenberg and Trevathan, 2002; Haig, 1993). Indeed, using robust human genome-wide association findings from the maternal genome for gestational duration and preterm birth risk, a recent study showed that preterm birth-associated regions harbored diverse evolutionary signatures, including those of accelerated evolution, evolutionary conservation, long term balanced polymorphism, and population differentiation (LaBella et al., 2020). In addition, control of the parturition cascade would be expected to diverge across species depending upon their reproductive ecology and environmental exposures. These divergent control mechanisms may also reflect the theoretical evolutionary advantages of different models we put forward above. For example, the Fixed Clock model could be more resistant to transient environmental or biological perturbations. The Parturition Block and Parturition Stimulation models, alternately, could enable sensing of changes during pregnancy and immediate response by feedback loops to optimize pregnancy in more dynamic fashion.

Evolutionary approaches that collect, curate, and compare the same pregnancy-related data across species promise to be particularly informative, especially to shed light into the innovations leading to possibly the re-wiring of regulatory network of preexisting genes not previously involved in parturition into pregnancy regulation. The reproductive strategy differences and consequences of ecologic niche and environmental exposures will further inform the mechanisms that prompt birth. We return to principal proposed by the Danish physiologist Krogh, 1929 that “For such a large number of problems, there will be some animal of choice or a few such animals on which it can be most conveniently studied’ to apply to parturition initiation. The ideal ‘animals of choice’ would have sequenced and annotated genomes, the ability to isolate and characterize blood and tissues through gestation in the mother and fetus, and informative phenotypes that could be together analyzed to reveal molecular pathways that determine the duration of gestation; at least for the species in question. Genome editing technologies could then be used for functional validation.

**Figure 5.**
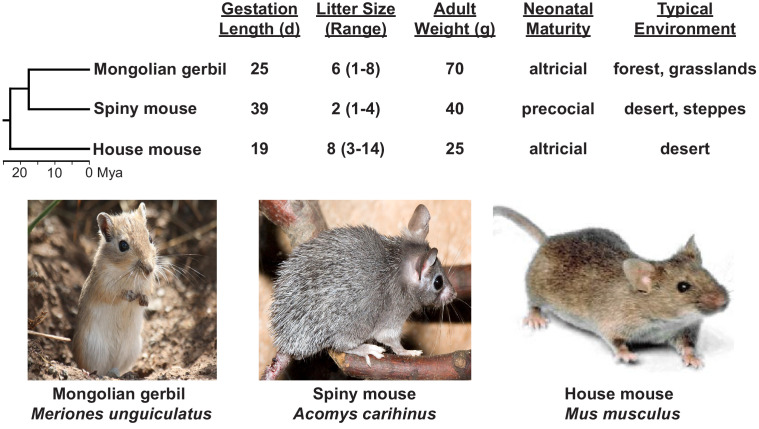
Muridae phylogeny and divergence times of the spiny mouse, Mongolian gerbil and house mouse with their associated pregnancy characteristics. Image of Mongolian gerbil is from https://en.wikipedia.org/wiki/Mongolian_gerbil#/media/File:Meriones_unguiculatus_(wild).jpg, image of spiny mouse from https://commons.wikimedia.org/wiki/File:Acomys.cahirinus.cahirinus.6872.jpg, and image of house mouse from https://en.wikipedia.org/wiki/House_mouse#/media/File:Mouse_white_background.jpg.

Considering these potential animals of choice in an evolutionary framework promises to be particularly revealing. We will provide one example in the Muridae family that could be explored. The spiny mouse (*Acomys carihinus*), Mongolian gerbil (*Meriones unguiculatus*), and house mouse (*Mus musculus*) have evolutionary divergence times from one another of 18–24 million years (Kumar et al., 2017; Figure 5). Remarkably, the spiny mouse has a gestation duration of 39 days and delivers precocial neonates (Haughton et al., 2016), while house mice (Murray et al., 2010) and Mongolian gerbils (Cheal, 1983) have gestational durations of approximately 19.3 days and 25 days, respectively, and deliver altricial neonates.

In Figure 5, we show other characteristics of these species relevant to pregnancy and birth timing. Importantly, each of these species has sequenced genomes (https://www.ncbi.nlm.nih.gov/nuccore/PVKX00000000.1, https://genome.ucsc.edu/cgi-bin/hgGateway, Cheng et al., 2019). Comparison of evolutionary signatures in these recently diverged species may reveal the genomic alterations that led to the prolonged gestation in the spiny mouse; these alterations can subsequently be functionally dissected through dense longitudinal -omics experiments across multiple tissues, genome editing, and other commonly used modern approaches. In this way, the key target molecules would be elucidated, potentially providing support for one of the models we describe. A similar strategy could be applied to other species, such as in anthropoid primates, which differ from Muridae in characteristics such as not showing systemic P4 withdrawal, having typically singleton pregnancies and longer gestational periods, but also where functional analyses are more challenging.

The ‘animal of choice’ evolutionary approach combined with recent evidence from human genome-wide association studies may reveal shared pathways, gene networks, transcription factors, and regulatory elements controlling human pregnancy and parturition. The initial gestation length loci identified by GWAS in the human maternal genome suggest critical roles of the decidua, the maternal interface with the developing placenta and fetus, and estrogen signaling in decidual cells (Zhang et al., 2017). For example, a noncoding variant in the *WNT4* gene region shown to be involved in the regulation of gestational duration in mothers has been identified to mechanistically contribute to birth timing by establishing a new high affinity estrogen receptor binding site (Zhang et al., 2017). Is the decidua gauging a developmentally regulated fetal signal or is it the maternal mechanism of the clock that is activated possibly as early as implantation? The answers to these questions are not known and provide an interesting foundation to test hypotheses with emerging data. Exciting new data on the role of the vaginal microbiome (Callahan et al., 2017) and the ontogeny of pregnancy immune system modulation (Aghaeepour et al., 2017) provide further high-dimensional data sets, and together with emerging metabolic studies provide unprecedented opportunity for testing novel hypotheses. For example, does the maternal or fetal genome, in the context of maternal microbiome, modulate maternal metabolism in a consistent fashion? Does this then shape birth timing and risk for preterm birth? Additionally, new informatic methods that integrate findings across multi-omics platforms have found enhanced predictive power in relation to individual omics platforms and also revealed novel interactions (Ghaemi et al., 2019).

## Concluding remarks

The determinant of birth timing is a critical and exciting big question that awaits elucidation. Situated at the intersection of biology, environment, and social context, including race and racism, preterm birth poses challenges that we must address. Are there other general models and frameworks for solving the enigma of birth timing? For example, an alternative framework and set of models would focus on the source of parturition initiation: maternal, fetal, interactive maternal-fetal, environmental, etc. The community of biomedical scientists interested in this area of investigation are encouraged to comment on this article with their ideas, constructively challenge one another, and us, and work to solve the problem. Further, one intent of this dialogue is to bring new voices and ideas into the discussion. We hope to stimulate the vision and curiosity of diverse investigators to gain new insights and answer this important question.
